# To CT or not to CT: Questioning the Cost-Effectiveness of CT Thorax in Head and Neck Cancers

**DOI:** 10.1007/s13193-022-01608-5

**Published:** 2022-08-04

**Authors:** Nawaz Usman, Preethi S. Shetty, Punit Singh Dikhit, Diksha Dinker, Naveena A. N. Kumar, Akhil Palod, Koteshwara Prakashini, Priya P. Sankaran, G. Somu

**Affiliations:** 1 Department of Surgical Oncology, Manipal Academy of Higher Education (MAHE), Manipal Comprehensive Cancer Care Centre, Kasturba Medical College, Manipal, Karnataka 576401 India; 2 Department of Radiodiagnosis and Imaging, Manipal Academy of Higher Education (MAHE), Kasturba Medical College Manipal, Manipal, Karnataka 576104 India; 3 Department of Hospital Administration, Kasturba Medical College Manipal, Manipal, Karnataka 576104 India

**Keywords:** CT thorax, Distant metastatic workup, CT scan, Computed tomography, Second primary tumor, Cost effectiveness

## Abstract

Computed tomography (CT) scan has been an integral part of the diagnostic workup for patients with head and neck squamous cell carcinoma. Our study was designed to find out the incidence of distant metastasis and second primary tumor and to correlate the cost-effectiveness of CT thorax in detecting the same. This study was conducted among 326 cancer patients who visited our center with curative intent in the year 2021, with lesions in various head and neck subsites. Data were collected based on their pathological TNM staging and the presence of distant metastasis as evident on their CT thorax imaging with various variables related to the disease. Incremental cost-effectiveness ratio (ICER) was calculated for detecting a single metastatic deposit and second primary tumor in terms of Indian currency and was correlated to each subsite and stage of disease at presentation. Out of these 326 patients, 281 patients were included in our study after considering the inclusion criteria, and among these 281 patients, 235 of them underwent CT thorax for metastatic workup. No patient was found to have a second primary. Metastases were found in 12 patients. The site of primary lesion and clinical tumor (cT) staging were found to be significantly influencing the incidence of metastasis on CT thorax. ICER was least for larynx, pharynx, and paranasal sinuses and was highest for oral cavity primaries and early-stage disease. As per our observations and results of ICER, CT thorax is indeed a valuable modality but should be used judiciously when it comes to initial diagnostic workup.

## Introduction

Squamous cell carcinoma of the head and neck still remains a major health-related problem, particularly so in South Asian countries, including India. Overall disease prognosis has not improved much over the years despite the introduction of new diagnostic modalities and new treatment strategies. Around 25% of deaths in HNSCC can be attributed to the second primary tumor (SPT) and distant lung metastasis (DM) [1]. The most common site for DM deposit from HNSCC is the lungs, with an incidence of around 8 to 15% as described in multiple clinical studies [2]. Primary lung cancers, which can be included under SPT, account for about 23% of patients with HNSCC [2]. Often, the treating surgeon comes across situations where radical curative intent surgery for locoregionally advanced cancer is ultimately a failure because of an SPT or a DM focus.

CT scan has drastically changed the treatment strategies in the new era and is widely used in the field of oncology to assess the primary site of head and neck cancer, nodal disease, and staging as a part of the established TNM staging system. CT thorax as an imaging modality is an extremely useful tool for assessing primary tumor, nodal metastasis, and distant metastasis. This widely available modality has given the surgeons the ability to prognosticate the patient during the initial screening itself.

An important question remains as to how often one needs to do pre- and postoperative screening for distant metastases. Although the National Comprehensive Cancer Network® (NCCN®) gives a picture regarding CT thorax frequency and indications, these blanket guidelines cannot be applied to all patients, especially the patients in the Indian subcontinent [3]. A preoperative chest X-ray is warranted in all cases as a part of a presurgical workup. If the primary tumor and nodal status place the patient at high risk for pulmonary metastasis, a preoperative computed tomography scan of the chest is indicated. As crucial as it may sound, in a developing country like India, it still contributes to burdening the vast majority of patients who belong to a poor socio-economic status along with the potentially deleterious effects of added radiation exposure. Henceforth, this study was conducted in order to evaluate the cost-effectiveness of CT thorax as a metastatic workup.

## Materials and Methods

The electronic medical records of patients were accessed for the demographic details, clinical staging, CT findings, and final tumor board decision. All patients above 18 years of age with biopsy-proven squamous cell carcinoma (SCC) of HNC attending the Oncology out-patient department (OPD) (i.e., Surgical Oncology, Radiation Oncology, and Medical Oncology) between the period January 2021 and December 2021 were screened for the inclusion and exclusion criteria (Table 1).

**Table 1:** Inclusion and exclusion criteria of the study

	Inclusion criteria	Exclusion criteria
Histopathology	Squamous cell carcinoma (SCC)	Other pathology, i.e., lymphoma, salivary gland tumor, and melanoma
Site	Oral cavity, lip, oropharynx, larynx, hypopharynx	Nasopharyngeal, unknown primary neck, thyroid
Primary/recurrent	Primary	Recurrent
Mode of imaging	CECT thorax	PETCECT, if done as primary scan

The CT thorax of the eligible patients was reviewed by two radiologists (one is a tumor board member and another independent senior radiologist) for the presence of pulmonary metastases, second primary lung, mediastinal nodes, and other site metastases. Pulmonary nodules more than 5 mm, without spiculated margins, smooth located peripherally, mediastinal lymph nodes >/= 10 mm, and solitary spiculated lesion centrally located >/= 10 mm were noted as metastatic. Histologic confirmation of metastasis was not mandatory.

**Fig. 1:**
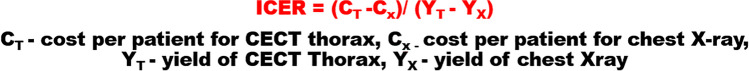
Computation of cost effectiveness of CECT thorax over Chest Xray using incremental cost effectiveness ratio (ICER)

The details were then analyzed using the SPSS software version 26. Factors influencing the incidence of metastasis were analyzed by logistic regression. For estimating the cost-benefit analysis, the absolute cost of CECT thorax and chest X-rays was obtained from the hospital billing section. The yield of each diagnostic modality was defined as the number of patients with metastasis divided by the number of patients going through the diagnosis strategy. Cost-effectiveness was measured using the incremental cost-effectiveness ratio (ICER) and was calculated as shown in Fig. 1. This estimates the additional cost per additional patient with metastasis detected by CECT thorax over chest X-ray.

## Results

A total of 326 patients with biopsy-proven HNSCC attended the Oncology OPD between January 2021 and December 2021 and were screened for eligibility for the study. Of these, 281 patients fit the aforementioned criteria. Amongst them, 235 patients had undergone CT thorax as a part of staging workup and, finally, 200 patients had locoregionally advanced clinical staging (AJCC 8th Edition Stage III & IV).

A total of 12 patients of the 200 (6%) were found to have unequivocal metastases on the CT thorax. Of these, 7 (59%) had pulmonary metastasis alone, 2 (17%) had liver metastasis alone, and one (8%) had bony metastasis alone. Two patients had multiple organ metastases, i.e., pulmonary + liver and liver + splenic metastases each. No patient was found to have a synchronous second primary. These 12 patients had a change in disease management from curative to palliative intent.

The average age of the patient with or without metastasis on CT thorax was similar (59.2 +/− 11.3 years vs 56.7 +/− 11.7 years, respectively). The incidence of metastasis on CT thorax was similar amongst the gender group too (6% for males and 5.9% for females). The highest incidence of metastasis on CT thorax was noted amongst smokers (12.1%), followed by tobacco chewer (7.7%) and alcohol intake (6.3%). There was no metastasis seen in patients who were purely betel nut chewers. Patients with no tobacco/alcohol intake had an incidence of metastasis at 3.2%.

The incidence of the metastases on CT thorax with respect to the site of primary, clinical T, and N staging has been elaborated in Table 2.

**Table 2:** Incidence of metastasis on CECT thorax based on clinical staging and site of primary

	Metastasis on CECT thorax		Total number of patients (*n* = 200)
	Present (*n* = 12)	Absent (*n* = 188)	
Age (in years)	59.2 +/− 11.3	56.7 +/− 11.7	*P*-value = 0.47
Gender			*P*-value = 1.00
Male	10 (6%)	156 (94%)	166
Female	2 (5.9%)	10 (94.1%)	12
Habits			*P*-value = 0.32
None	2 (3.2%)	60 (96.8%)	62
Betel nut chewing	0	21 (100%)	21
Alcohol	2 (6.3%)	30 (93.8%)	32
Tobacco chewing	4 (7.7%)	48 (92.3%)	52
Smoking	4 (12.1%)	29 (87.9%)	33
cTumor stage			*P*-value = 0.028
Early (T1, T2)	1 (2.9%)	33 (97.1%)	34
Advanced (T3, T4)	11 (6.6%)	155 (93.4%)	166
cNodal stage			*P*-value = 0.471
N0	1 (1.9%)	52 (98.1%)	53
N+	11 (7.5%)	136 (92.5%)	147
Site of primary			*P*-value = 0.012
Oral cavity and lip	4 (3.6%)	106 (96.4%)	110
Oropharynx	1 (14.3%)	6 (85.7%)	7
Larynx	4 (22.2%)	14 (77.8%)	18
Hypopharynx	2 (3.3%)	58 (96.7%)	60
Paranasal sinus	1 (20%)	4 (80%)	5

On univariate analysis, the site of the primary lesion and cT staging was found to be significantly influencing the incidence of metastasis on CT thorax (*p* = 0.012 and *p* = 0.028, respectively). Clinical N staging was not found to be significantly affecting the development of metastasis. Furthermore, on multivariate and logistic regression analysis, cT staging alone was found to be significant (HR–4.22. CI: 1.07–16.54, *p*-value = 0.039).

The CECT thorax was evaluated against a chest X-ray for cost-benefit analysis (Table 3).

**Table 3:** Cost benefit analysis of CECT thorax vs chest X-ray

	CECT thorax		Chest X-ray		ICER
	Cost per patient (*C*_T_)	Yield (*Y*_T_)	Cost per patient (*C*_x_)	Yield (*Y*_X_)	(*C*_T_ – *C*_x_)/(*Y*_T_ – *Y*_X_)
All patients	Rs 7000	0.06	Rs 200	0	Rs 113,333
cTumor stage					
T1/2	Rs 7000	0.03	Rs 200	0	Rs 226,666
T3/4	Rs 7000	0.07	Rs 200	0	Rs 97,142
cNodal stage					
N0/1	Rs 7000	0.04	Rs 200	0	Rs 170,000
N2/3	Rs 7000	0.07	Rs 200	0	Rs 97,142
Site of primary					
Oral cavity and lip	Rs 7000	0.04	Rs 200	0	Rs 170,000
Oropharynx	Rs 7000	0.14	Rs 200	0	Rs 48,571
Larynx	Rs 7000	0.22	Rs 200	0	Rs 30,909
Hypopharynx	Rs 7000	0.03	Rs 200	0	Rs 226,666
Paranasal sinus	Rs 7000	0.20	Rs 200	0	Rs 34,000

The average cost of a CECT thorax was Rs 7000 per patient, and that of a chest X-ray was Rs 200 per patient. The chest X-ray did not pick up any metastasis, while the yield of CECT thorax was 6%. Based on this, an ICER of Rs 113,333 was noted. This represents the additional cost of using CECT thorax over chest X-rays to identify one additional patient with metastasis. On subgroup analysis, cT stage, location of the primary site was found to significantly affect the ICER. The ICER was least for cT3/4 lesions (Rs 97,142 per patient) and doubled for cT1/2 lesions. Similarly, their ICER was significantly lower for patients with oropharyngeal, laryngeal, and paranasal sinus primaries compared to the oral cavity and hypo-pharyngeal primaries.

## Discussion

The aim of our study was to evaluate the cost-effectiveness of CT chest during initial screening to search for SPT and DM foci, especially in financially constrained situations as seen in India. These guidelines, emphasizing the need for CT chest in newly diagnosed HNSCC, have not been evaluated in terms of cost-effectiveness and subsite-based analysis. We came across multiple studies on detection rates of CT scans for SPT and DM. A study by Ong et al. where significant emphasis to detect SPT and DM was given on the CT chest in initial screening involved around 47% of patients with laryngeal malignancies while only 28% of patients belong to the oral subset [4].

Another study by Reiner et al. to evaluate the role of CT thorax in detecting DM foci and SPT in patients with HNSCC involved 189 patients, of which 63 patients had laryngeal primaries, 53 had pharyngeal primaries and 72 had oral cavity primaries. Out of these patients, SPT was detected on the CT chest in 4 patients with laryngeal malignancies and 6 patients with pharyngeal malignancies, with no evidence of SPT in oral cavity subsets, while DM was found in 6 patients with laryngeal malignancies, 4 patients of pharyngeal malignancies, and 11 patients of oral cavity subsets, all of which were stage IV disease. Although this aforementioned study was designed to evaluate the efficiency of chest X-ray in detecting lung abnormalities as compared to CT chest, and they showed that CT thorax was a superior modality, they did not evaluate the cost-effectiveness of CT scan for detecting single SPT or DM [1].

Similar findings were suggested by Fukuhara et al. where the supremacy of CT chest as compared to chest X-ray was advocated but the primary subset of patients was again laryngeal malignancies, with oral cavity subsets being only about 16% of the total study group. They were able to detect 23% of lung nodules (SPT or DM), which changed the treatment plan, thus advocating CT thorax during initial screening but missing on the part of cost-effectiveness based on head and neck subsites and staging [5].

The rate of detection of distant metastasis at initial presentation ranged between 1.5 to 20% [6, 7]. As compared to these studies, in our study data the incidence of DM was around 6% while there was no SPTs. A study by Nagarkar et al. reported findings similar to ours, with the incidence of DM being 3.2% while not picking up SPTs [8]. Advanced T and N stages were found to significantly correlate with the rate of pickup of DMs. This is also echoed in the NCCN guidelines that mention CT thorax as a metastatic work-up in higher T and N stages [3]. We noted a significant association between cT stages and subsite of the primary tumor, while N status failed to correlate significantly. These discrepancies warrant the need for reassessing the need for CT thorax as a routine distant metastatic workup for all HNSCC patients.

The incidence of DM in head and neck cancers is relatively small compared with other malignancies like stomach, pancreas, lung, or breast [7]. This range of variation in the incidence of DM can possibly be explained by the fact that all these studies have different numbers of populations of primary index tumors.

Interestingly, in our study as well as in the aforementioned studies, the larynx was a common site associated with increased risk of DM and SPT as compared to oral cavity subsites. If this associated difference was because of any particular habit history or any other risk factor was beyond the scope of our study.

NCCN guidelines recommend CT scan in locoregionally advanced HNSCC^3^. However, part of the justification for this recommendation has been the claim that CT also has the additional advantage of picking SPTs, but on careful evaluation of the literature, we found that the incidence of SPT was low at 1.5% in the study by Fukuhara et al. [5] and 5.7% among 1086 patients in a study by Shah et al. [9]. Even in an extensive literature review by Warren and Gates, where a total of 1259 cases were studied in detail, a total incidence of 3.5% of SPT was noted. In our data of 281 patients, we did not find even a single case of SPT, thereby questioning the claim that CT thorax has the added advantage of picking up SPTs [10].

We did a cost analysis based on our findings and calculated the ICER, which showed differences in cost-effectiveness based on subsites. This difference was most pronounced in the oral cavity subsite and early-stage tumor and for N0/N1 stage disease. The superiority of a CT chest over a conventional X-ray has been established beyond doubt and this is reflected in the NCCN guidelines where preoperative CT chest has been advised during initial workup to look for DM, SPT, and mediastinal lymphadenopathy [3]. However, in a country like ours, there is a need to justify the additional financial burden this approach entails. Our study data and results reflect a way that can be used for modifying existing guidelines. The majority of the patient population in our study comprised of the oral cavity subsite, which contributed to only around 3.6% of the total detected DMs, while laryngeal cancers contributed to 22% of the total detected DMs. Interestingly, all the patients in oral cavity subsites who had metastatic deposits (4 patients) belong to locoregionally advanced stages.

The NLST trial showed the benefit of CT scan screening in people with a high risk of lung cancer (one of their inclusion criteria was cigarette smoking for 30 pack years) [11, 12]. We only had smoking as a habit in 16.5% of our patients. And only 2 of them smoked 30 pack years. This could explain why we did not find any second primaries in our study.

On cost effectiveness analysis, the ICER was most efficient for subsites like larynx, oropharynx, and paranasal sinuses and for locoregionally advanced disease. Our analysis showed that the ICER of performing CT scan across all comers was a hefty 133,000 Indian rupees. With increasing cost of health care services, every screening procedure must be justified not only based on sensitivity but cost efficiency as well.

Luke Tan et al. published a study to evaluate the benefit of CT chest as a screening tool in patients with HNSCC. A total of 20 patients were included in the study, and they concluded that even after adding an additional cost of $13,314, CT chest did not add to the extra sensitivity for detecting DM and SPT [13].

Although the efficiency and sensitivity of CT chest for detecting DM and SPT are proven and have been included in internationally accepted guidelines, the blanket use of CT chest for all HNSCC patients is not cost-effective as per our experience. We found a statistically significant correlation in terms of ICER between DM and cT4 oral disease with a value of 170,000/−, for oropharynx and hypopharynx 48,571/− and 226,666/− respectively, for larynx 30,909/− and for paranasal sinuses 34,000/−. Another factor that had a favorable ICER in our study was a nodal disease, i.e., N0/N1 disease vs N2/N3 disease, but this difference was statistically insignificant.

## Conclusion

In developing countries with limited resources and a high burden of disease, it is the need of the hour to cut down on non-cost-effective diagnostic modalities. Although our study design and findings are not robust enough to make rigid recommendations, they do suggest that there is a scope to make CT thorax as a diagnostic modality more cost-effective. CT thorax can be used in locally advanced HNSCC as a part of staging workup; however, our cost analysis has not supported use in all the stages and all subsites. The need of the hour is to design larger, preferably prospective studies so that we can identify the subsets that benefit most from CT scan as a screening modality for metastases and avoid them in the rest, thereby decreasing the financial burden on the healthcare and also decreasing the deleterious effects of radiation exposure.
